# Supplementary material for: Effects of shady environments on fish collective behavior
Source: Sci Rep. 2022 Oct 25;12:17873. doi: 10.1038/s41598-022-22515-3 (PMC9596401; doi:10.1038/s41598-022-22515-3)
Supplement: Supplementary file 1 — Supplementary Figures. [file 41598_2022_22515_MOESM1_ESM.pdf]

Effects of shady environments on fish collective behavior

Haroldo V. Ribeiro[1,*], Matthew R. Acre[2,†], Jacob D. Faulkner[2], Leonardo R. da Cunha[1], Katelyn M. Lawson[3], James J. Wamboldt[4], Marybeth K. Brey[4], Christa M. Woodley[5], and Robin D. Calfee[2]

[1]Departamento de Física, Universidade Estadual de Maringá, Maringá, PR 87020-900, Brazil
[2]U.S. Geological Survey, Columbia Environmental Research Center, 4200 New Haven Road, Columbia, Missouri, 65201, USA
[3]Department of Biological Sciences, Auburn University, 101 Rouse Life Sciences Building, Auburn, Alabama, 36849, USA
[4]U.S. Geological Survey, Upper Midwest Environmental Sciences Center, 2630 Fanta Reed Road, La Crosse, Wisconsin, 54603, USA
[5]U.S. Army Corps of Engineers, Engineer Research and Development Center, 3909 Halls Ferry Road, Vicksburg, Mississippi, 39180, USA
[*]email: hvr@dfi.uem.br
[†]email: macre@usgs.gov

Supplemental Materials

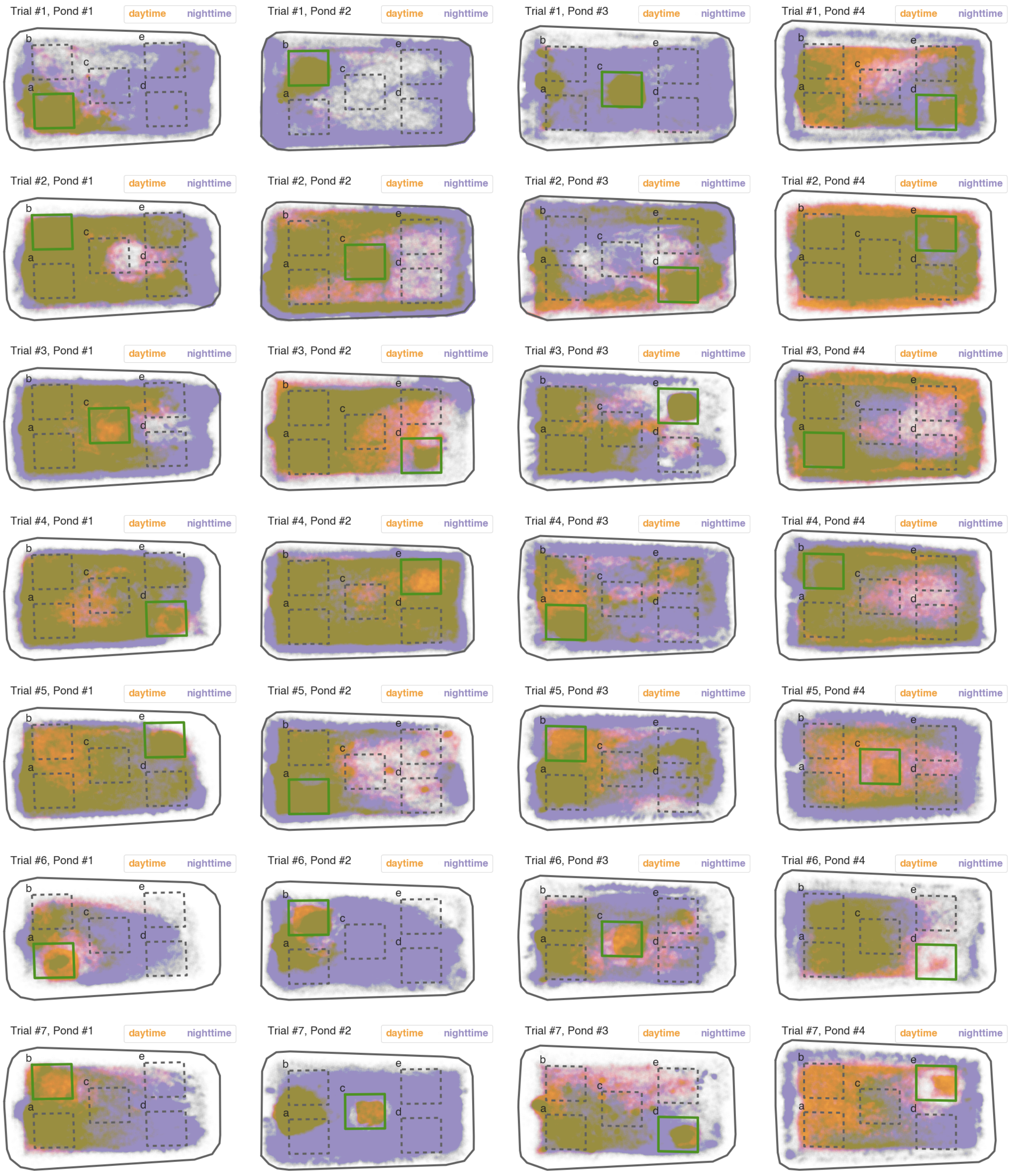

Figure S1. Density maps of fish positions from each pond and trial used in our study. Orange hues indicate positions recorded during daylight hours, while purple hues represent positions collected at night. The gray solid lines represent the pond limits and the thick green squares indicate the actual location of the shade structure in each trial and pond (as shown in the top left of the panels). The dashed squares indicated by lowercase letters (from 'a' to 'e') represent all possible shade positions. The shade structures are respectively located at positions 'a', 'b', 'c', and 'd' of ponds 1, 2, 3, and 4 in trial 1. These shade structures are then sequentially moved over the trials; for instance, the shade locations over the trials in pond #2 are: 'c' → 'd' → 'e' → 'a' → 'b' → 'c' → 'd'.

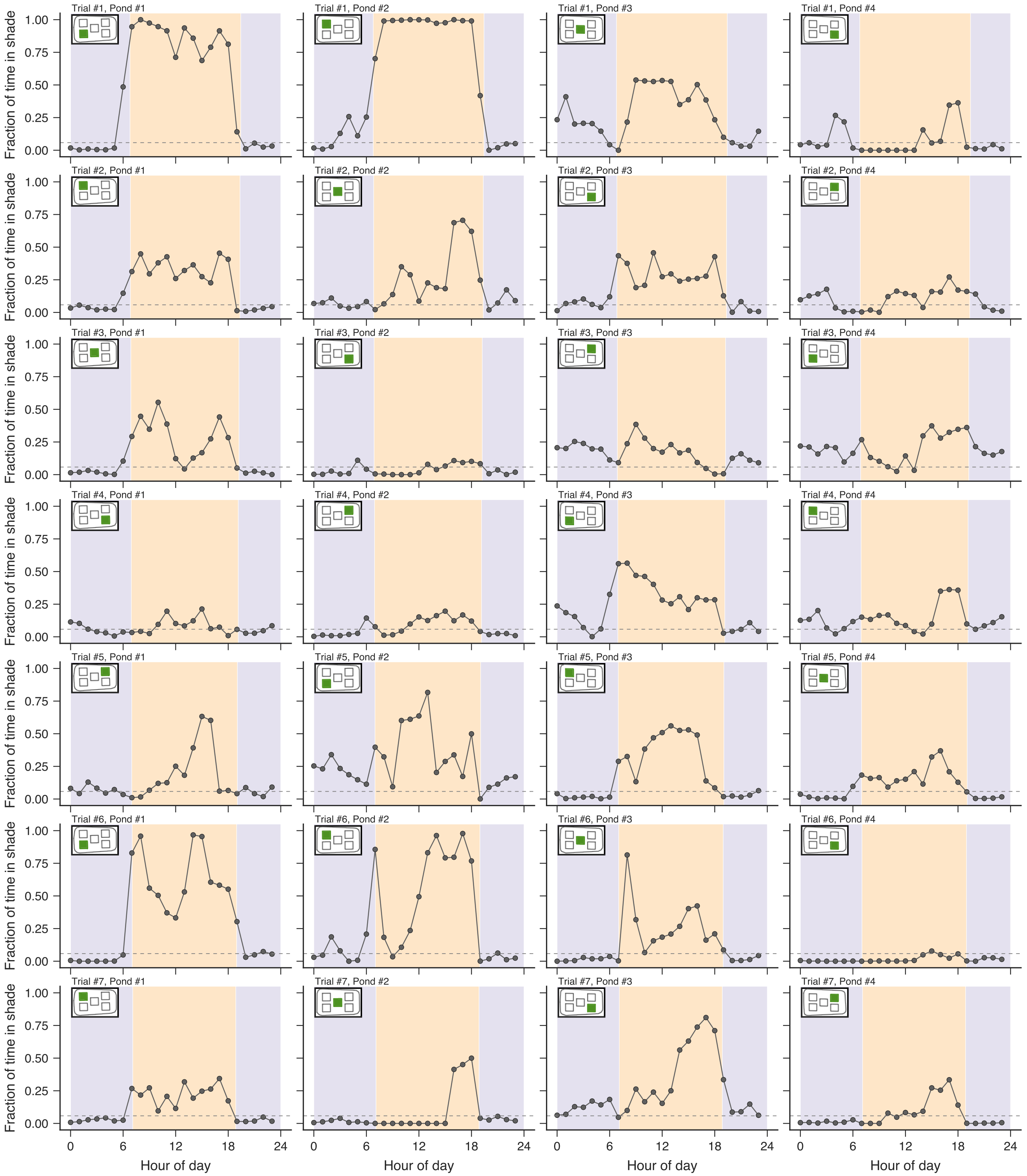

Figure S2. Differences in shade selection as a function of the hour of day in each pond and trial. The circles show the fraction of all fish positions recorded under the shade structures within a one-hour time window for each pond and trial (as shown in the top left of the panels). The dashed line represents the expected fraction of positions under shade if fish would move randomly over the ponds, and the background colors indicate daytime (orange) and nighttime (purple). Small insets in each panel show the pond and shade location in each trial and pond.

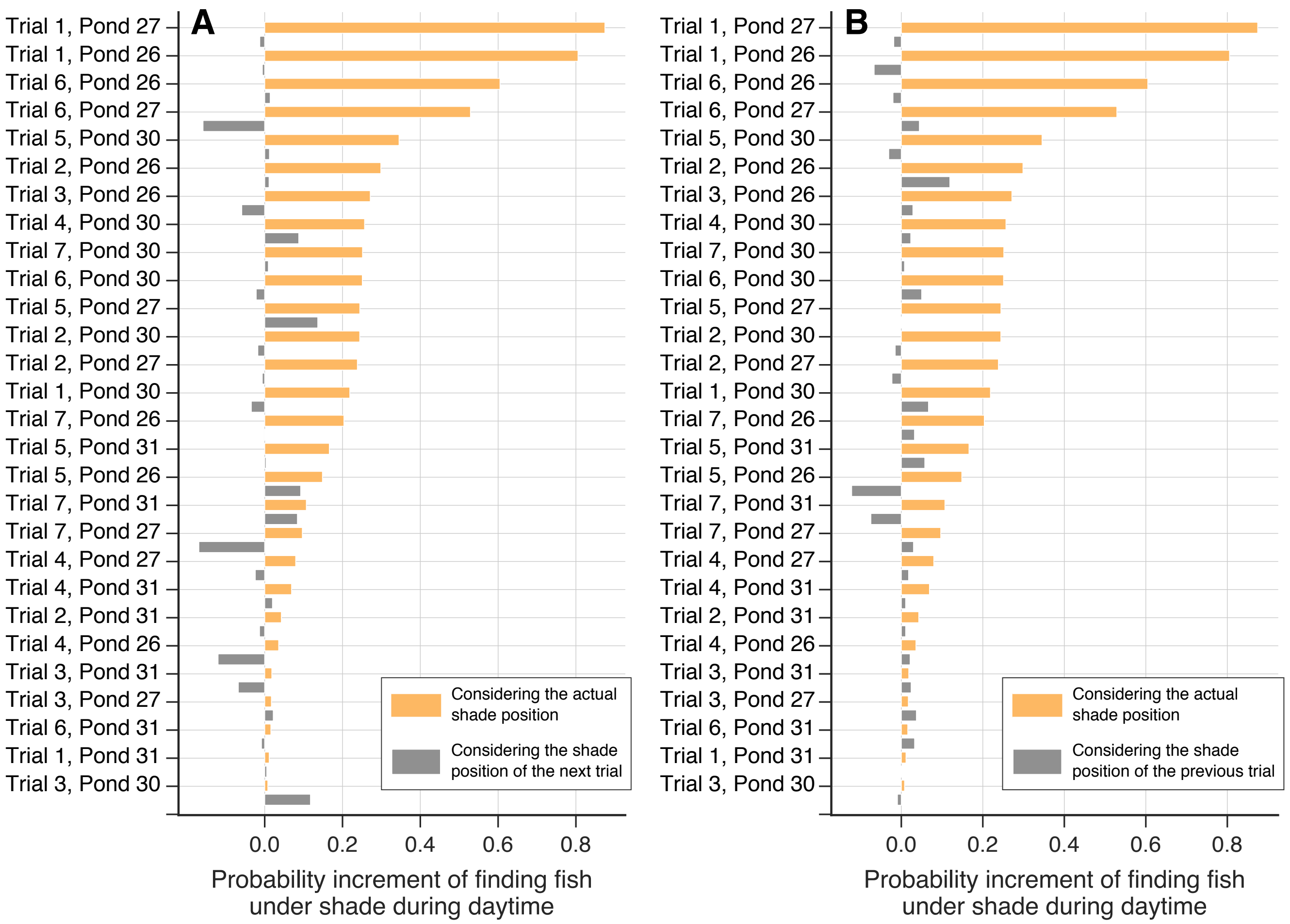

Figure S3. Probability increment of finding a position under a shade structure during daylight hours compared with a control-like experiment that considers the shade location of the next and previous trials. These probabilities are estimated via logistic regression for each trial and pond. The orange bars show the values considering the actual shade location while the gray bars depict the probabilities when considering the shade location of the next trial (Panel A) and the previous trial (Panel B) in each pond. The relationship between a fish selecting shade or not the binary independent variable of day or night vanishes when not considering the actual shade location.

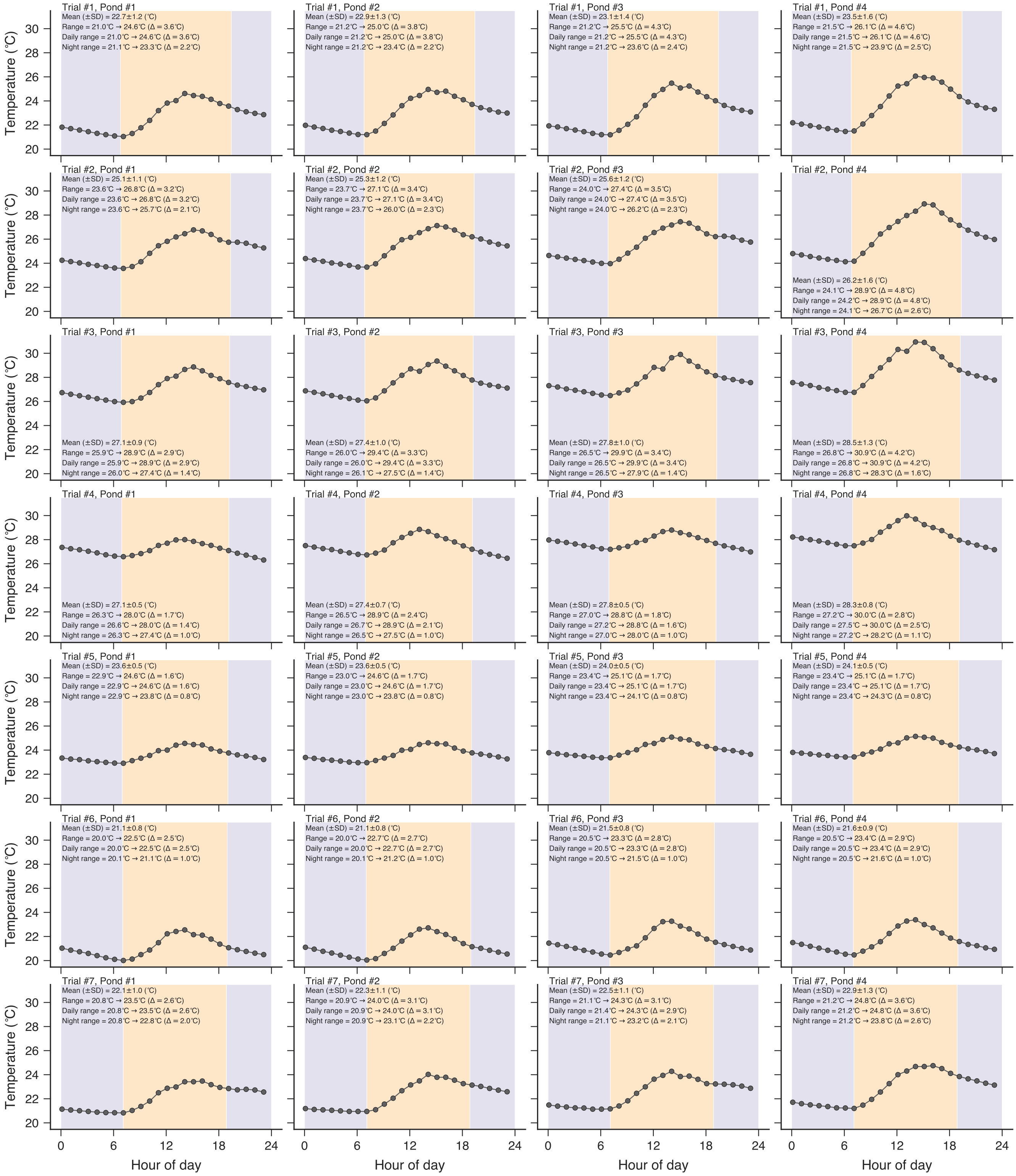

Figure S4. Average water temperature as a function of the hour of day in each pond and trial. The circles show the average water temperature within a one-hour time window for each pond and trial (as shown in the top left of the panels). The background colors indicate daytime (orange) and nighttime (purple). Descriptive statistics related to average temperature are shown in the top left of the panels. The overall average temperature varied from ≈21° to ≈29°. The daily range of average temperature varied from ≈1.5° to ≈5° , while the night range of average temperature varied from ≈1° to ≈2.5°.

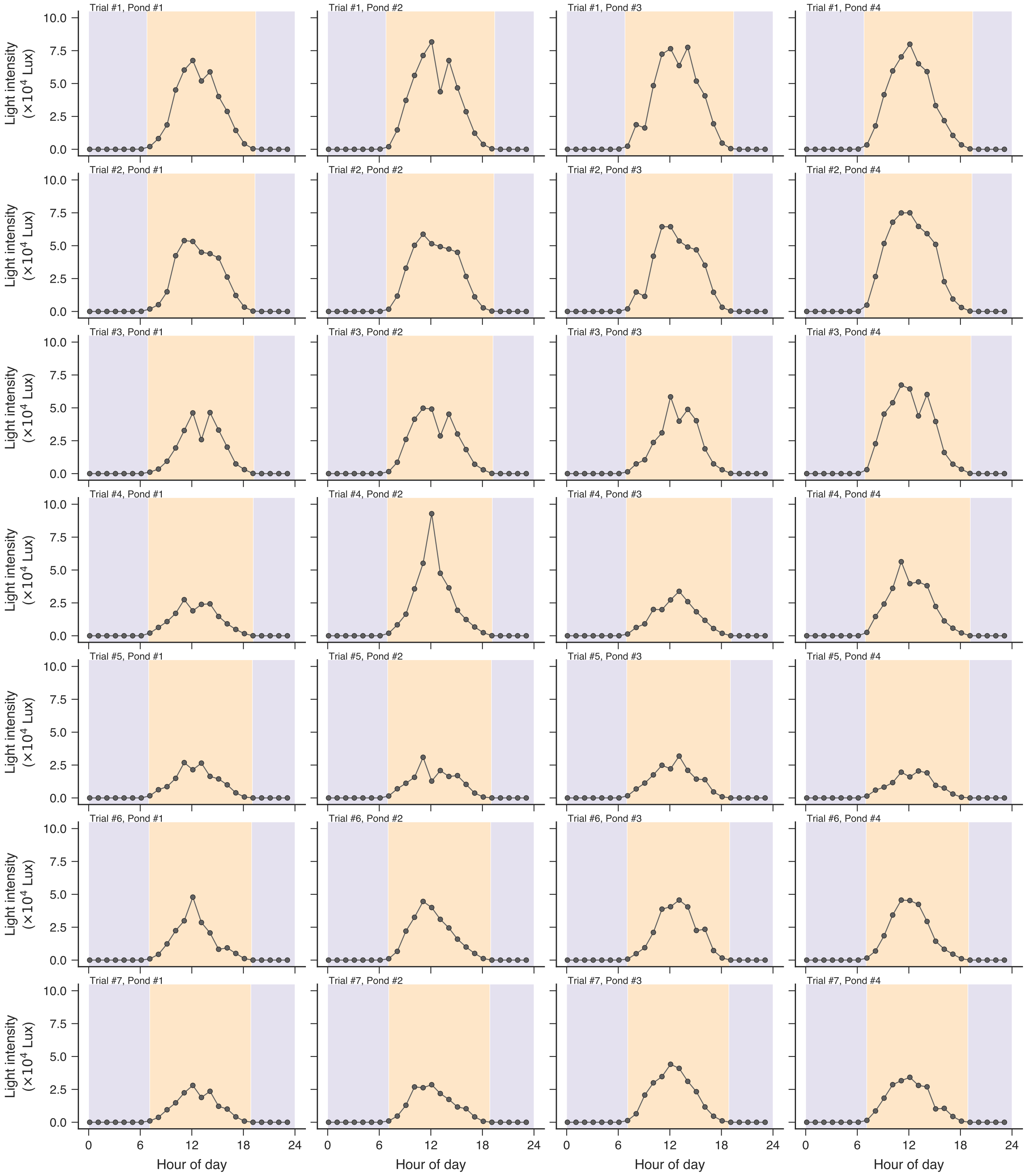

Figure S5. Average light intensity as a function of the hour of day in each pond and trial. The circles show the average light intensity within a one-hour time window for each pond and trial as monitored by an underwater sensor positioned on the north end of each pond. Light intensity increases fast after sunrise, reaches a peak around noon, and approaches zero with the sunset.

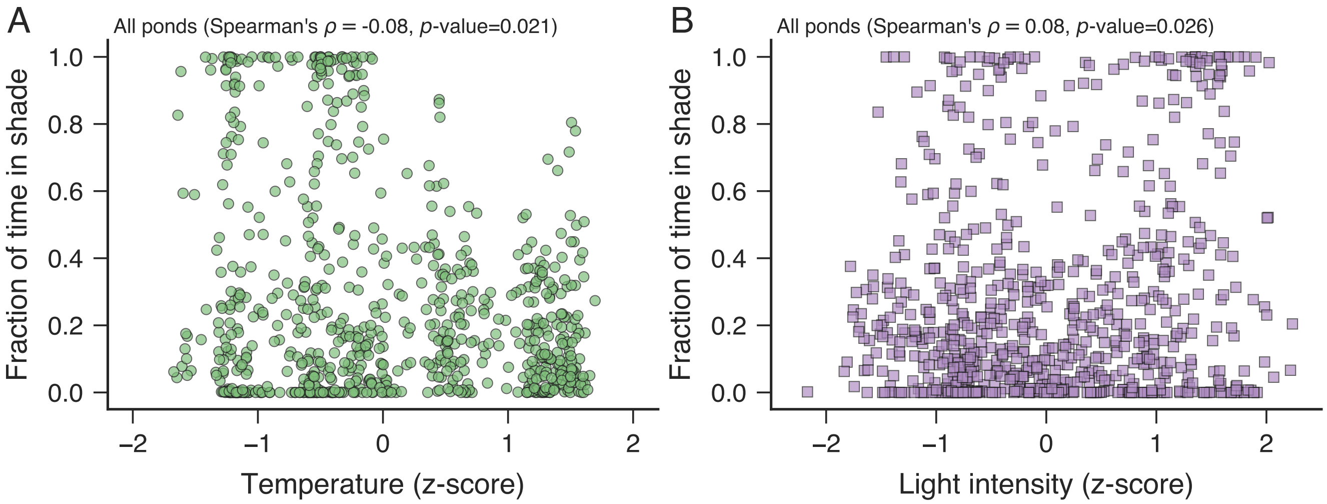

Figure S6. Shade selection is not well correlated nor exhibits discernible patterns with water temperature and light intensity. (A) Relationship between the fraction of all fish positions recorded under the shade structures during the day within a one-hour time window and average water temperature. (B) Relationship between the fraction of all fish positions recorded under the shade structures during the day within a one-hour time window and average light intensity. The predictive variables (water temperature and light intensity) are in standard score units (z-scores), that is, raw values subtracted from their hourly averages and divided by their hourly standard deviations over the trials in each pond. There is no clear association between the fraction of time in shade and the temperature (Spearman's $\rho = -0.08$, p-value$= 0.021$) as well as between the fraction of time in shade and the light intensity (Spearman's $\rho = 0.08$, p-value$= 0.026$).

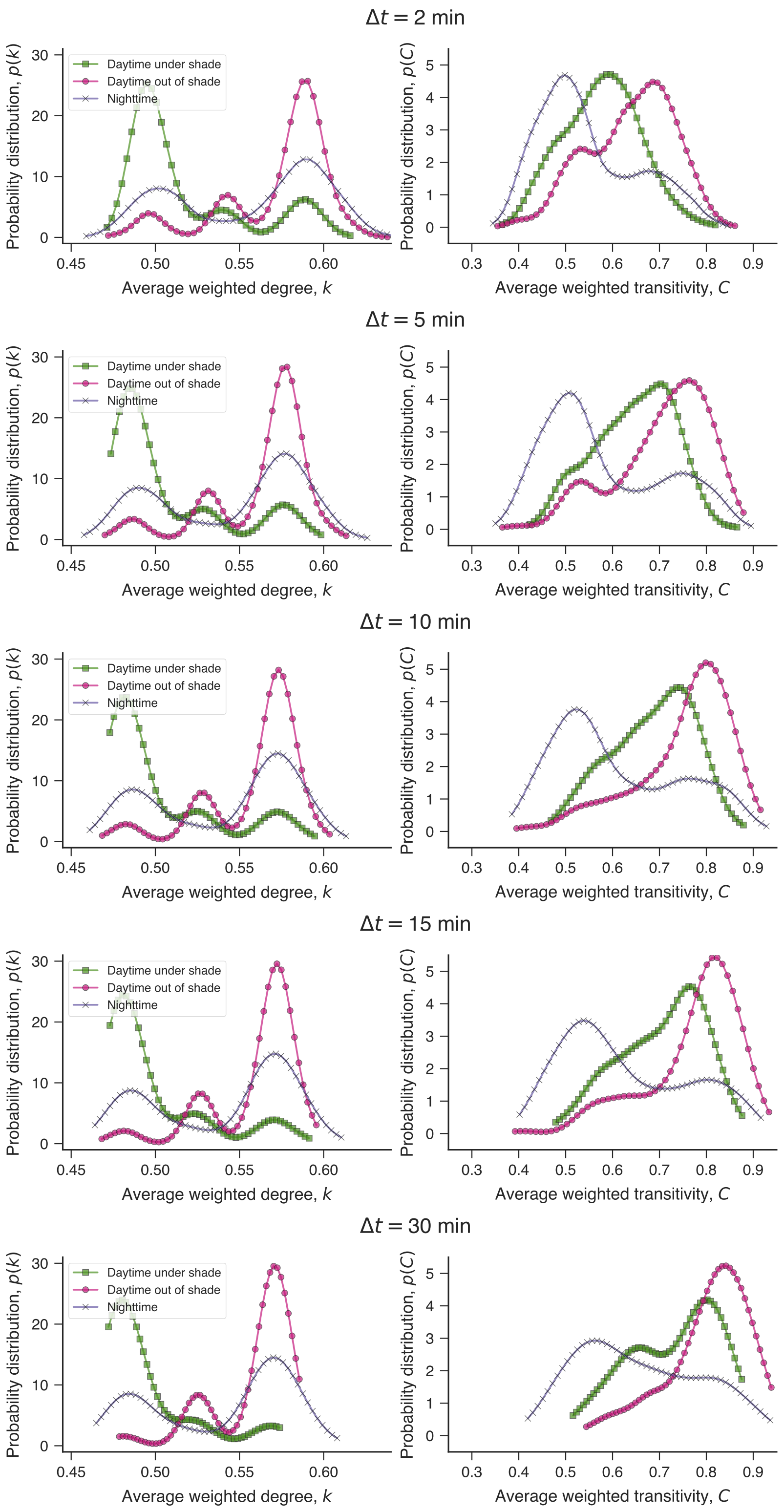

Figure S7. Sensitivity analysis at five time intervals of network measures. Each row of plots shows the probability distributions of the same network measures depicted in Figure 4 of the main text at the various time intervals Δt used to create the weighted networks (values are indicated within the plots). We note that these distributions do not differ significantly among the different time intervals Δt.

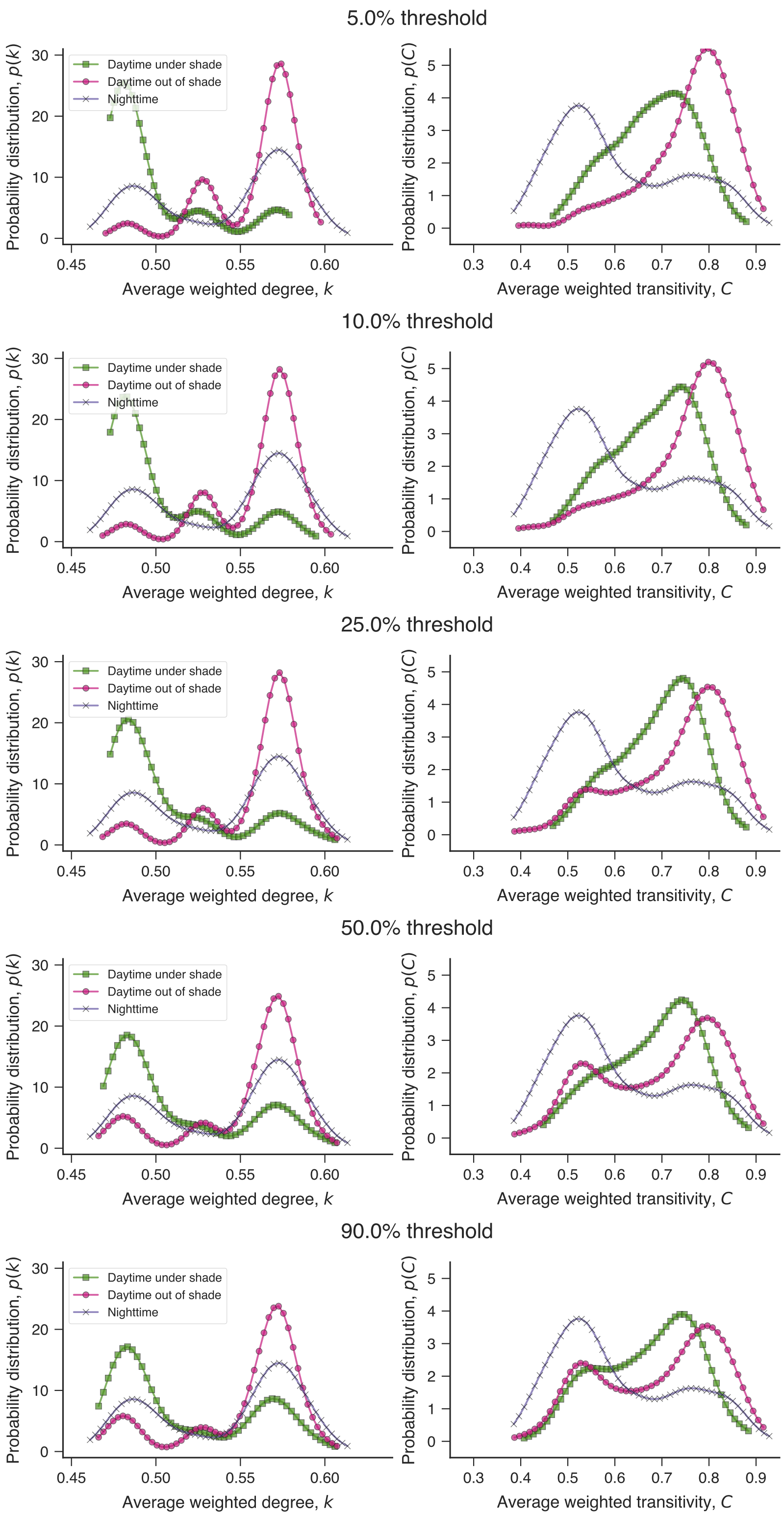

Figure S8. Sensitivity analysis at five thresholds of network measures used to define the under-shade category in the analysis related to the interaction networks. Each row of plots shows the probability distributions of the same network measures depicted in Figure 4 of the main text for various thresholds (values are indicated within the plots). We note that these distributions do not differ significantly among the different thresholds.
